# Doctor-Patient Cancer Screening Communications among Church-based Chinese Adults – The Role of Caregiver Experience and Family History

**DOI:** 10.31557/APJCP.2021.22.1.241

**Published:** 2021-01

**Authors:** Su-I Hou

**Affiliations:** *Department of Health Management & Informatics, College of Community Innovation and Education, University of Central Florida, Orlando, FL, USA.*

**Keywords:** Cancer screening communication, Chinese, caregiver, family history

## Abstract

**Objective:**

Significant gap exists in the literature examining cancer screening communication related factors among Chinese immigrants. This study examined the role of cancer caregiver experience and family history on doctor-patient cancer screening communication among church-based Chinese adults.

**Methods:**

A self-administered survey was conducted among adults from 9 Chinese churches (n=372). Cancer Communication was measured by “Dr. recommended screenings” and “Talked to doctors about cancer screenings”. The survey was developed in English and translated in Chinese.

**Results:**

Mean age was 44.31 (SD=14.74), 60% were males, 72% were married, majority had college education (85%), and 17% reported had been a primary cancer caregiver and 54% reported having family cancer history. Cancer caregivers scored higher on doctor-patient cancer communication, as well as cancer knowledge and screening norms. Participants with family cancer history were also more likely to talk to doctor about screening, as well as perceived higher cancer risk, lower health status, and screening barriers. Multiple regression analyses showed that primary caregiver experience was still a significant predictor on talking to doctors about cancer screenings (OR=2.1; 95%CI=[1.10, 4.01]; p=0.025), yet doctors more like to recommend screening among caregivers became non-significant. The significant influence of family cancer history on talking with doctors on cancer screenings also disappeared. Older age (OR=2.52; p=0.006) and being married (OR=2.45; p=0.022) were significant on predicting communication of cancer screenings with doctors. Data also showed that doctors were more likely to recommend cancer screenings to older (OR=2.75, p<.001), married (OR=2.57; p=0.006) adults.

**Conclusion:**

Current study calls attentions to primary cancer caregiver experience, family history, age, and marital factors when designing tailored doctor-patient cancer screening communication programs among church-based Chinese to address cancer disparities.

## Introduction

*Rationales for conducting cancer communication study in Chinese Immigrants*

Chinese-American is the largest Asian subpopulation (23.0%), with over two-thirds as first-generation immigrants born overseas (ACS, 2016). However, current research show Chinese-American is under-represented in cancer studies and reported low screening adherence. With complex barriers towards cultural, language, and healthcare access, Chinese immigrants face deepened disparities in cancer screening participations (Sentell et al., 2015; Hou, 2010). Faith-based organization has a unique position to deliver health and social services to the hard-to-reach communities (Hou and Cao, 2018a; Hou and Liu, 2018b; Hou, 2016a; Hou, 2016b; Holt et al, 2009). However, there is a significant gap in the literature examining Chinese or Chinese-American in faith-based organizations overall. Majority of the existing faith-based cancer interventions have been conducted among African Americans (Griffith et al, 2012; Emerson et al., 2009; Holt et al., 2009) and Latino Americans (Schwingel and Galvez, 2016; Allen et al, 2014; Colon-Otero et al., 2014), yet few have focused among Asian immigrants overall (Hou and Cao, 2019; Hou and Cao, 2018a). Significant gap also exists in the literature examining doctor-patient cancer screening communication among Chinese immigrants. 

The role of primary cancer caregiver experience on doctor-patient cancer screening communication 

Limited studies have examined doctor-patient cancer communication among cancer caregivers and results have been inconsistent. Studies show while caregivers had higher cancer awareness (Elangovan et al., 2016), caregivers reported having limited cancer knowledge and very low screening self-efficacy (Nasiriani, et al., 2017; Anusha and Nagarathnam, 2016). A study in Iran found only 13% of the caregivers were screened for mammography, over 70% of those who were not screened indicated “the doctor did not prescribe for me” as the main reason, and near 90% did not know when or how to perform breast self-exam (Naririani et al., 2017). 

There has been overwhelming evidence showing provider recommendation significantly improves screening rates (Peterson et al., 2016). Family cancer caregivers desire cancer information and communication with doctors (Hou 2016a; Longacre et al., 2015). However, studies on cancer communication with doctors examining the role of caregivers have been limited, either within the U.S. or overseas. The few existing studies show primary care physicians are not recommending screenings among caregivers at a satisfactory level, and a significant portion of caregivers had at least one or more unanswered screening concerns (Lin et al., 2016). 

*The role of family cancer history on doctor-patient cancer screening communication*

The link between family cancer history and higher cancer risk has been confirmed by scientists and researchers (CDC, 2019; Ramsey et al., 2006). However, existing studies find those with family cancer history or high risk of cancers are still uninformed of their cancer risk or never received a physician recommendation for screening (Salimzadeh et al., 2016; Fletcher et al., 2007). A large U.S. colorectal cancer study among patients with family history showed almost half did not know they should be screened at a younger age (Fletcher et al., 2007). Similarly, another large colorectal cancer study in Iran examined first degree relatives (FDRs) of cancer patients also showed low screening rate and knowledge, and low awareness of their own increased cancer risk (Salimzadeh et al., 2016). These unsatisfactory findings may at least partially due to the fact that general physicians need to be educated to better perform their gatekeeper role and answer screening related questions among patients with family cancer history (Lunsford et al., 2018; Rose et al., 2001). Rose and colleague studied physician’s knowledge on referring patients with family history of cancers showed that primary care doctors have difficulty in deciding whether to refer due to limited genetic knowledge. Most general physicians prefer referring only moderate and high risk, but not low risk patients for screenings (Rose et al., 2001). 

*Gap on the role of caregiver and family history on doctor-patient cancer screening communications among Chinese adults*

Although there have been a number of studies focusing on the mental or physical health among cancer caregivers (Warapornmongkholkul et al., 2018; Lee et al., 2017; Nipp et al., 2016; Sklenarova et al., 2015). There are limited studies examining doctor-patient cancer screenings communication in light of the role of caregivers or those with family history (Polek and Hardie, 2016). Existing limited literature pointed while caregivers might view cancers less fatalistic, their overall cancer knowledge and communication are low. Doctor-patient cancer screening communication deserves greater attention, especially among the under-studied Chinese or Chinese immigrant groups. The current study examines the role of cancer caregiving experience and family cancer history on doctor-patient screening communication among church-based Chinese adults. The author hypothesizes that primary cancer caregivers and those with family history will be more likely to have doctor-patient cancer communication, as well as score higher on cancer knowledge and beliefs. Given a lack of literature focusing on doctor-patient cancer screening communication and the role of caregiver and family history, and with the strength of a mixed sample of Chinese-immigrants in the U.S. and overseas, this study fills in the important gaps in the literature on both the subjects and populations, as well as providing critical implications to healthcare professionals interacting with Chinese patients.

## Materials and Methods

*Design and Sample*

The current study was a cross-sectional study assessing doctor-patient cancer screening communication and examining the role of caregiver and family history among church-going Chinese adults. A convenience sample of five major Chinese churches in a large metropolitan city in the Southeastern U.S., and four Chinese churches in a large metropolitan city in Taiwan participated in the study (total n=372) (Hou and Cao, 2019; Hou and Liu, 2018b). This convenient sample was chosen given existing established relationship with the researcher. Strategies used to recruit study participants included informing key church leaders, making formal announcements at the Sunday services, setting up tables in high-traffic areas such as the sanctuary foyer, lobby, cafeteria, as well as visiting small group gatherings. About 80% of the Chinese adults invited voluntarily participated (n=372). All the study churches provided mandarin services as their main services. Reasons for possible non-participation could be due to lack of time or interests, or other obligations at the time of the survey. The study was approved by the human subject office at the investigator’s institution.

In order to detect meaningful difference on doctor-patient cancer communication, as well as cancer knowledge and beliefs, by caregiver experience (yes / no) or family cancer history (yes / no), minimum sample size needed were calculated. Mean differences and standard deviations were estimated from earlier published results (Hou and Cao, 2019; Hou and Liu, 2018b), using 5% alpha level, 80% detecting power, and 1:1 ratio between two groups, minimum sample sizes ranged between 21 to 63 per group in these comparisons. The current study sample met the minimum sample size to have 80% power to detect meaningful differences between groups.

*Measurement*

Study questionnaire was developed in English, translated into Chinese, and back-translated to ensure consistencies and accuracy of item meanings (Hou et al., 2014; Hou and Chen, 2004; Hou et al., 2003). The self-administered survey took 10-15 minutes to complete. A small thank-you gift was provided for each participant. 

Cancer communications were measured by two self-reported survey items, “Dr. recommended screenings” and “Talked to doctors about cancer screenings” to capture the two-way communication dimensions. Both the cancer caregiver experience and family cancer history were yes / no questions asking whether participants had “ever been a primary caregiver for a family member”, and whether participants “had any relatives or family members who had been diagnosed with cancers.” 

Author’s 8-item validated Cancer Screening Knowledge Test (CSKT) (Hou and Liu, 2018b; Hou et al., 2014; Hou, 2016c) and a 14-item Cancer Warning Signs Test (CWST) (Hou and Liu, 2018b) were used to assess objective cancer knowledge. Both scales were developed in collaboration with community partners and stakeholders, as well as pilot tested among multiple Chinse samples in Taiwan with satisfactory reliabilities and validities (Hou and Liu, 2018b; Hou, 2016c; Hou et al., 2014). The Cronbach alphas of CSKT and CWST scales were .701 and .909 respectively. CSKT consisted of a good mix of eight (8) easy and moderate difficult items measuring key cancer risk factors, need for regular screenings, recommended guidelines of major age-appropriate cancer screenings, and cancer early signs. The CWST measured the seven (7) cancer warning signs (C.A.U.T.I.O.N.) endorsed by the World Health Organization, as well as seven (7) non-cancer warning signs (Hou, 2016c). Each knowledge item answered correctly was coded as “1,” and coded as “0” otherwise. Possible scale ranges were 0-8 for CSKT and 0-14 for CWST. One research-tested subjective cancer knowledge item using 5-point Liker scale was used to measure participants’ subjective cancer knowledge level (Hou and Liu, 2018b; Hou, 2016c; Hou et al., 2014). The subjective knowledge item was a five-point liker scale item, with 5 being very high and 1 being very low. 

A previously validated theory-based Cancer Screening Belief Scale (CSBS) was also used in the current study (Hou, 2007). The domains for the CSBS include 3-item perceived screening benefits (pros), 4-item perceived screening barriers (cons), and 3-item perceived norms (norms). The reliabilities of these sub-scales were satisfactory with Cronbach alphas of 0.818 (CITC range, 0.652 to 0.689) for CSBS-Pros, 0.649 (CITC range, 0.343 to 0.497) for CSBS-Cons, and 0.722 (CITC range, 0.411 to 0.648) for CSBS-Norms (Hou, 2007). All items were measured via 5-point Likert scales. Possible ranges were 1-15 for CSBS-pros and CSBS-norms, and 1-20 for CSBS-cons. This scale has been tested among multiple Chinese sample with consistent satisfactory evidence of validities and reliabilities (Hou and Liu, 2018b; Hou, 2016a; 2016b; 2016c; Hou et al., 2014). 

*Analyses*

Descriptive analyses were used for demographic variables. Chi-square tests were used to compare the two cancer communication variables by primary cancer caregiver and family cancer history. T-tests were used to compare continuous variables including subjective and objective cancer knowledges, cancer screening beliefs scales between groups. Multiple logistic regressions were used to exam how caregiver experience and family cancer history predicted the two cancer communications variables, adjusting for demographics co-variables including age, gender, marital status, education, and country.

## Results

*Participant characteristics*

A total of 372 Chinese adults from nine churches participated. About half of the participants lived in the U.S and half lived in Taiwan. Overall 62% of the participants aged 40+ years, 60% were males, 72% married, and majority had college education (85%). Overall 17% reported had been a primary caner caregiver, and over half of the study participants reported family cancer history (54%). There were no significant differences between country sites by age, marital status, family cancer history, or talk to doctor about screenings (Hou and Cao, 2019). 

*Cancer communications, knowledge, and screening beliefs by cancer caregiver experience*

Study also found that older (49.9 vs. 43.2 years; p=.002) and male Chinese adults (73.3% vs. 57.0%; p<.05) were more likely to be cancer caregivers. Results showed that those who had been cancer caregivers were also more likely to talk with doctors about cancer screenings (48.3% vs. 23.4%, p<.001) and report doctors recommended cancer screenings to them (56.7% vs. 31.8%, p<.001). Analyses also showed that those who had been a primary cancer caregiver to a family member were more likely to score higher on perceived cancer knowledge (3.33 vs. 2.73, p<.001), cancer screening knowledge test (6.16 vs. 5.78, p=.030), cancer warning sign test (6.95 vs. 5.97, p=.036), and perceived cancer screening norms (11.95 vs. 11.12, p=.004). 

*Cancer communications, knowledge, and screening beliefs by family cancer history*

Current data show that Chinese adults with family cancer history tended to be older (46.4 vs. 41.9 years; p=.004) and married (78.8% vs. 64.1%; p=.002). Findings showed that participants with family cancer history were more likely to report talked with doctors on cancer screenings (32.2% vs 21.5%; p=.025), and scored higher on cancer warning sign tests (6.43 vs. 5.70; .034) and lower on perceived cancer screening barriers (10.40 vs. 11.19, p=.011). Data also showed those with family cancer history were more likely to perceived higher cancer risk (2.78 vs. 2.53; p=.003) and lower health status (3.14 vs. 3.46; p=.001). Although findings indicated doctors were also more likely to recommend those with family cancer histories to screen for cancers, the difference was not statistically significant (39.7% vs 30.7; p=.079). There wer, however, no significant differences on doctors recommending screenings, subjective cancer and screening knowledge by family cancer history. 

*Multiple logistic regression analyses*

After controlling for demographics (age, gender, marital, and education) along with country difference (USA vs. Taiwan), multiple logistic regression analyses showed that primary caregiver experience to a family member was still a significant predictor on talking to doctors about cancer screenings (OR=2.1; 95%CI=[1.10, 4.01]; p=.025), yet doctors more like to recommend screening among caregivers became non-significant. The significant influence of family cancer history on talking with doctors on cancer screenings also disappeared after controlling for key demographics and country. Oder age (OR=2.52; p=.006) and being married (OR=2.45; p=.022) were also significant on predicting communication of cancer screenings with doctors. Data also showed that doctors were more likely to recommend cancer screenings to older (OR=2.75, p<.001), married (OR=2.57; p=.006) adults. There were no significant differences on cancer communications by gender or education. 

**Table 1:** Cancer Communications, Knowledge, & Screening Beliefs by Primary Cancer Caregiver Experience

Variables	Had been a primary Caregiver (n=63) Mean (SD)	Not a primary cancer Caregiver (n=309) Mean (SD)	p-value
Dr. recommend screenings	56.70%	31.80%	<0.001**
Talked with dr. on screenings	48.30%	23.40%	<0.001**
Subjective cancer knowledge (1-item)	3.33 (.951)	2.73 (.906)	<0.001**
Cancer Screening Knowledge Test (8-item)	6.16 (1.089)	5.78 (1.2589)	0.030*
Cancer Warning Sign Test (14-item)	6.95 (3.070)	5.97 (3.189)	0.036*
CSBS-Pros (3-items)	12.92 (2.136)	12.44 (2.096)	0.11
CSBS-Cons (4-items)	10.53 (3.365)	10.81 (2.874)	0.515
CSBS-Norms (3-items)	11.95 (1.861)	11.12 (2.058)	0.004*

CSBS, Cancer Screening Belief Scale (CSBS)

**Table 2:** Cancer Communications, Knowledge, & Screening Beliefs by Family Cancer History

Variables	Had Family History (n=201) Mean (SD)	No Family History (n=171) Mean (SD)	p-value
Dr. recommend screenings	39.70%	30.70%	0.079
Talked with dr. on screenings	32.20%	21.50%	0.025*
Subjective cancer knowledge (1-item)	2.91 (.914)	2.73 (.953)	0.061
Cancer Screening Knowledge Test (8-item)	5.89 (1.435)	5.74 (1.654)	0.36
Cancer Warning Sign Test (14-item)	6.43 (3.186)	5.70 (3.157)	0.034*
CSBS-Pros (3-items)	12.59 (2.106)	12.43 (2.098)	0.467
CSBS-Cons (4-items)	10.40 (2.963)	11.19 (2.882)	0.011*
CSBS-Norms (3-items)	11.43 (1.807)	11.03 (2.287)	0.072

**Table 3:** Multiple Regressions of Caregiver Experience and Family Cancer History on Cancer Communications, Adjusting for Demographics (Age, Gender, Marital, and Education) and Country

DV	Dr. recommend screenings		Talked with dr. on screenings	
	X^2^_ (7)_ =52.89 (p<.001); 67.8%		X^2^_ (7)_ =41.38 (p<.001); 73.7%	
IV	OR [95% CI]	p-value	OR [95% CI]	p-value
Cancer Caregiver (Yes)	1.90 [.989, 3.632]	0.054	2.10 [1.095, 4.014]	0.025*
Family History (Yes)	1.05. [.635, 1.735]	0.851	1.21 [.706, 2.063]	0.493
Age (>=40)	2.75 [1.530, 4.942]	<0.001**	2.52 [1.311, 4.828]	<0.006**
Marital (married)	2.57 [1.303, 5.077]	0.006*	2.45 [1.135, 5.268]	0.022*
Education (college+)	0.95 [.464, 1.953]	0.894	1.06 [.497, 2.275]	0.875
Gender (Male)	1.47 [1.469, .890]	0.133	1.22 [.717, 2.074]	0.464
Country (Asia)	1.56 [.937, 2.612]	0.087	1.40 [.811, 2.410]	0.228

Reference groups: Caregiver (no); Family history (no); Country (U.S.); Age (<40); Marital (single); Education (<=high school); Gender (Female)

## Discussion

Two previously published articles also analyzed data from the current study sample (Hou and Cao, 2019; Hou and Liu, 2018b). One focused on objective versus subjective knowledge by younger versus older age groups (Hou and Liu, 2018b). The other one compared cancer knowledge and beliefs among participants from different country, USA vs. Taiwan (Hou and Cao, 2019). To provide deeper insights on the complex factors critical for tailored cancer screening communication intervention development, this study conducted further analyses to examine the role of primary caregiver experience and family cancer history on doctor-patient cancer screening communications specifically. Relevant results from previously published articles were integrated.

Current data show primary cancer caregivers were more likely to report doctor recommended screenings (56.7% vs. 31.8%), communicated with doctor about screenings (48.3% vs. 23.4%), scored higher on cancer knowledge and warning signs, perceived higher cancer knowledge levels and screening norms (all p<.05). Chinese adults with family cancer history were more likely to communicate with doctors about screenings (32.2% vs. 21.5%; p<.05) and perceived higher cancer risk (2.78 vs. 2.53; p=.003), lower health status (3.14 vs. 3.46; p=.001) and lower screening barriers (10.39 vs. 11.19; p=.011). After controlling for demographics and country, regression analyses showed that caregiver experience was still a significant predictor on cancer communication with doctors (OR=2.1). Yet, the significant relationship of talk with doctors on screenings among those with family history disappeared.

Current study found that experience of being a primary cancer caregiver to a family member was significant to initiate communications with doctors about screening. This could be resulted from the increased awareness and knowledge towards cancer prevention, which were supported with current data and previous studies (Elangovan, et al, 2016; Polek et al., 2016). To shed some lights on the comparisons and findings, the previously published results found that Taiwan participants were more likely to have been a cancer caregiver (22.9% vs. 10.4%; p=0.001) (Hou and Cao, 2019). In the specific analyses by country, Taiwan participants also scored higher on both objective cancer education knowledge scales: CSKT (mean of 6.13 vs. 5.52; p<0.001) and CWST (mean of 6.80 vs. 5.38; p< 0.001). Taiwan participants also rated higher on perceived (subjective) cancer knowledge (3.09 vs. 2.59; p< 0.001), and endorsed higher on CSBS-Norms-3 (mean of 11.67 vs. 10.82; p<0.001) (Hou and Cao, 2019). The higher knowledge scores and more positive beliefs among Taiwan participants were consistent to the findings of the higher knowledge and beliefs found among primary caregivers in the current finding. The higher portion of caregiver from the Taiwan participants might partially contribute to these higher scores among the caregiver group. Nevertheless, the current regression analyses did included country as a co-variate, and still found caregiver was a significant predictor towards talking to doctors about cancer screenings.

However, attentions should be given to those with family cancer history. Current results showed there were no significant differences of family history on cancer communications with doctors, doctors recommending screenings, nor cancer screening knowledge or related beliefs. Potential factors that might contribute to the non-significant findings could be either doctors didn’t ask or patients didn’t mention about family history, or other communication related barriers. More studies should also examine reasons why Chinese with family cancer history perceived higher cancer risk and lower health status, yet still had the same low communication rate with doctors about screenings.

Doctor recommendation is one of the most consistent predictors of adherence to screening cancer screening (Jih et al., 2018). Consistent with the study hypotheses and recommended age-appropriate screening guidelines, current data showed that doctors were 2.45-2.52 times more likely to recommend screenings to those who were at older age (>=40 years) or married. The previously published study which examined cancer knowledge by age provide some insights on potential underlying reasons (Hou and Liu, 2018b). While there were no significant differences between those who were older (>=40 years) versus younger (<40 years) on the objective cancer knowledge tests (CSKT and CWST), subjective knowledge was higher among older Chinese adults (younger.2.44 vs. older 3.05, p<.001) (Hou and Liu, 2018b). Older Chinese adults were also more likely to identify cancer warning signs correctly, while younger adults were more likely to identify false warning signs correctly. Studies have found that subjective knowledge to be a significant predictor on health behaviors (Hou and Liu, 2018b), thus the higher subjective knowledge among older Chinese participants might played role to the increased patient-doctor communication seen among older participants in the current findings.

The overall low doctor-patient cancer screening communication rate in the current finding is alarming. Existing studies show Chinese immigrants may face not only language barriers, but also cultural and other issues during the communication process (Jih et al., 2018; Kwok et al., 2011). Mixed methods research (MMR) which uses both quantitative and qualitative approaches to exam doctor-patient cancer communication might provide deepened and nuanced insights on some of the underlying reasons and barriers. 

Study is limited to a convenience sample among faith-based Chinese adults whom self-selected to participated in the study. In addition, study sample consisted of middle-age Chinese adults mostly highly educated yet with English as their second language, thus results should be interpreted with such sample characteristics in mind, and not generalizing to other Chinese adult groups. The mixing of US churches with the Chinese church overseas participants, along with a deeper and specific examination of the current study focus provide a unique opportunity to further explore the complex relationships among doctor-patient communication and the role of cancer caregiver and family history. 

Although sample size calculation showed sufficient power (80%) to detect meaningful differences between groups, it should be noted that only a relative small proportion of the current sample (17%) reported having been a primary cancer caregiver (n=63). In addition, current study used one yes/no question to identify primary cancer caregiver experience. Additional dimensions related to the caregiver experience, such as lengths of time as a caregiver, might need to be considered in future research. 

One strength of the current study was using items to capture the two-way doctor-patient communication dimensions, “Dr. recommended screenings” and “Talked to doctors about cancer screenings.” The current study compared own doctor-patient cancer screening communication by the role of primary cancer caregiver and cancer family history. Future research could further exam the context or scenarios of a primary cancer caregiver or those with a family cancer history talking to doctor about a family cancer communication, instead of cancer communication about own case.

*Practical Implications*

The current study is among the first to note the significant relationship of primary cancer caregiver experience on increased cancer screening communications with doctors among Chinese church-going adults. Although data show caregivers were more likely to proactively communicate with doctors on screening, findings showed alarming finding that there was no significant difference on doctor’s screening recommendations by family cancer history. This could possibly be the fact that not all individuals with family cancer history might need more frequent screenings. However, findings also point to the potential missed opportunities for needed screening referrals and the critical need of doctor training for culturally sensitive communication and screening this higher risk group (Lunsford et al., 2018; Rose, et al., 2001).

In conclusion, current study calls attentions to primary cancer caregiver experience, family history, age, and marital factors when designing tailored doctor-patient cancer communication programs among Chinese immigrants. Given the alarming overall low rate of doctor-patient cancer communication and high reported family cancer history (54%) among the current the church-going highly educated Chinese study sample, study shed light on the potential to further exam how primary caregiver experience might serve as an active engagement factor to promote positive doctor-patient cancer screening communications. 

Future studies using mixed-methods research (MMR) study designs are encouraged to provide nuanced insight on how the different dimensions of the caregiver experience, doctor-patient cancer screening communication, and how their experience might shape own cancer screening decisions. Similarly, MMR studies are recommended to exam concerns doctors might have when deciding whether or when to recommend screening for Chinese patients with family cancer history. 

## Statement conflict of Interest

The authors declare there is no conflict of interest.
